# Pannexin 1 Influences Lineage Specification of Human iPSCs

**DOI:** 10.3389/fcell.2021.659397

**Published:** 2021-04-16

**Authors:** Rebecca J. Noort, Grace A. Christopher, Jessica L. Esseltine

**Affiliations:** Division of BioMedical Sciences, Faculty of Medicine, Memorial University of Newfoundland, St. John’s, NL, Canada

**Keywords:** human pluripotent stem cells, pannexin channels, germ lineage specification, differentiation, CRISPR-Cas9 gene ablation

## Abstract

Every single cell in the body communicates with nearby cells to locally organize activities with their neighbors and dysfunctional cell-cell communication can be detrimental during cell lineage commitment, tissue patterning and organ development. Pannexin channels (PANX1, PANX2, and PANX3) facilitate purinergic paracrine signaling through the passage of messenger molecules out of cells. PANX1 is widely expressed throughout the body and has recently been identified in human oocytes as well as 2 and 4-cell stage human embryos. Given its abundance across multiple adult tissues and its expression at the earliest stages of human development, we sought to understand whether PANX1 impacts human induced pluripotent stem cells (iPSCs) or plays a role in cell fate decisions. Western blot, immunofluorescence and flow cytometry reveal that PANX1 is expressed in iPSCs as well as all three germ lineages derived from these cells: ectoderm, endoderm, and mesoderm. PANX1 demonstrates differential glycosylation patterns and subcellular localization across the germ lineages. Using CRISPR-Cas9 gene ablation, we find that loss of PANX1 has no obvious impact on iPSC morphology, survival, or pluripotency gene expression. However, *PANX1* gene knockout iPSCs exhibit apparent lineage specification bias under 3-dimensional spontaneous differentiation into the three germ lineages. Indeed, loss of PANX1 increases representation of endodermal and mesodermal populations in *PANX1* knockout cells. Importantly, *PANX1* knockout iPSCs are fully capable of differentiating toward each specific lineage when exposed to the appropriate external signaling pressures, suggesting that although PANX1 influences germ lineage specification, it is not essential to this process.

## Introduction

Pluripotent stem cells (PSCs), including embryonic stem cells (ESCs) and induced pluripotent stem cells (iPSCs) are characterized by the ability to self-renew indefinitely and the capacity to differentiate into theoretically any cell type in the embryo (Nagy et al., 1993; Itskovitz-Eldor et al., 2000). Differentiation is the process by which stem cells are assigned a cell fate. At each successive stage of lineage commitment cells become more and more specialized and lose the capacity to become other cell types. Terminal differentiation refers to the final stage of cell fate specification, when a cell is locked in form and function. One of the first cell fate decisions in development occurs during gastrulation when the embryo patterns the three embryonic germ layers: ectoderm, endoderm, mesoderm. The ectoderm eventually gives rise to tissues including skin and brain while the endoderm patterns for internal organs and the mesoderm contributes to muscle, bones, and connective tissues. Embryonic germ layer specification can be mimicked *in vitro* through active or passive differentiation paradigms of human PSCs.

Pluripotent stem cells (and epiblast cells in the embryo) maintain their stemness through activation of POU5F1, NANOG, and transforming growth factor beta (TGFβ) signaling pathways (reviewed in Tam and Loebel, 2007). Gastrulation and the subsequent emergence of the three germ layers from pluripotent stem cells initiates when Activin/Nodal, bone morphogenic protein (BMP), and WNT signaling pathways are activated (Camacho-Aguilar and Warmflash, 2020). WNT3 signaling enables formation of the primitive streak and specification of the bi-potent mesendoderm (Tada et al., 2005; Tam and Loebel, 2007; Tan et al., 2013). The mesoderm subsequently specifies through additional WNT3 pathway activation and BMP signaling, whereas endoderm is specified by elevated activation of Nodal/Activin A signaling pathways (Tam and Loebel, 2007; ten Berge et al., 2008). The ectoderm germ lineage is formed when remaining epiblast cells that did not ingress through the primitive streak are subjected to TGFβ/Nodal and BMP pathway inhibition (Tam and Loebel, 2007; Tchieu et al., 2017). Germ lineage specification can be mimicked *in vitro* through exogenous exposure to the aforementioned signaling molecules in directed differentiation of PSCs. In contrast to directed differentiation, spontaneous differentiation enables passive, cell-guided lineage commitment and generally results in cell populations from all three germ layers (Desbaillets et al., 2000; Itskovitz-Eldor et al., 2000; Takahashi et al., 2007).

Cells do not undertake differentiation in isolation; rather communication among neighboring cells, and between cells and the niche, is essential to ensure appropriate cell fate specification (Perrimon et al., 2012). In addition to secreted factors, small signaling molecules released from cellular channels also play increasingly recognized roles in these processes. For example, purinergic signaling through release of extracellular ATP participates in neural precursor cell and mesenchymal stem cell self-renewal, migration, and differentiation (Glaser et al., 2014; Cavaliere et al., 2015; Kaebisch et al., 2015; Jiang et al., 2017). Pannexin proteins (PANX1, PANX2, and PANX3) form large-pore channels, permeable to ions and metabolites less than 1 kDa in size (Penuela et al., 2013). PANX1 is widely expressed across multiple tissues in the body while PANX2 and PANX3 are more restricted in tissue expression (Penuela et al., 2013). The most well-defined role of PANX1 is ATP release (Chekeni et al., 2010). In healthy cells PANX1 channels normally remain in a closed state until induced to transiently open in response to a variety of stimuli including membrane deformation, receptor activation, and intracellular calcium release (Sandilos et al., 2012; Bao et al., 2013; Furlow et al., 2015; Sanchez-Arias et al., 2019). PANX1 signaling can also influence the self-renewal and differentiation of multiple somatic (adult) stem cell types including osteoprogenitor cells, skeletal myoblasts, and postnatal neural precursor cells (Wicki-Stordeur et al., 2012; Wicki-Stordeur and Swayne, 2013; Wicki-Stordeur et al., 2016; Ishikawa and Yamada, 2017; Pham et al., 2018). However, much less is known about the impact of PANX1 signaling in the early embryo or in pluripotent stem cells (Hainz et al., 2018).

Recent reports have revealed that PANX1 is highly expressed at the earliest stages of human development, and localizes to the plasma membrane of human oocytes as well as 2- and 4-cell stage human embryos (Sang et al., 2019). The high expression of PANX1 in human oocytes and embryos suggests a fundamental role for PANX1 in human development (Esseltine and Laird, 2016; Shao et al., 2016; Hainz et al., 2018). Indeed, several human disease-causing germline *PANX1* variants have now been identified. The first human patient identified to harbor a homozygous genetic variant in *PANX1* (PANX1-R217H) suffers from a staggering number of maladies in several of the organs most highly enriched in PANX1, including severe neurological deficits and primary ovarian failure (Shao et al., 2016). This mutation was shown to decrease PANX1 channel function while not affecting trafficking. Recently, four independent families were reported in which different heterozygous *PANX1* variants cause female infertility due to primary oocyte death (Sang et al., 2019). These four human variants interfered with PANX1 posttranslational modification and plasma membrane trafficking, decreased PANX1 protein abundance in cells, and aberrant channel function.

Because PANX1 is expressed at the very earliest stages of human development, and because human mutations negatively impact human oocyte survival, we sought to uncover whether PANX1 also impacts human pluripotent stem cells or stem cell fate decisions. Here we find that PANX1 protein is expressed at the cell surface of human iPSCs. *PANX1* knockout (*PANX-/-*) iPSCs generated through CRISPR-Cas9 gene ablation appear morphologically indistinguishable from control. Interestingly, we find enhanced representation of endodermal and mesodermal cells from spontaneously differentiated *PANX1-/-* iPSCs compared to control. Therefore, we conclude that PANX1 protein expression influences PSC commitment to the three embryonic germ layers.

## Materials and Methods

### Induced Pluripotent Stem Cell Lines

All studies were approved by the Human Ethics Research Board (HREB # 2018.210). Female wildtype human induced pluripotent stem cells (iPSCs) were created from dermal fibroblasts isolated from an apparently healthy 30-year-old female as described in Esseltine et al. (2017) and obtained through a material transfer agreement with The University of Western Ontario.

iPSCs were routinely cultured as colonies in feeder-free conditions in a humidified 37°C cell culture incubator buffered with 5% CO_2_ and atmospheric oxygen. iPSCs were grown on dishes coated with Geltrex (Cat# A141330, ThermoFisher, Waltham, MA, United States) and fed daily with Essential 8 medium (Cat# A1517001, ThermoFisher). Colonies were passaged every 4-5 days when they exhibited tight cell packing, smooth borders, and phase-bright smattering at colony centers. Individual iPSCs within the colonies exhibited prominent nucleoli and high nucleus-to-cytoplasm ratio as is characteristic for human pluripotent stem cells (Thomson et al., 1998; Takahashi et al., 2007). For passaging, iPSCs were incubated with gentle cell dissociation buffer (Cat# 13151014, ThermoFisher) at room temperature until colonies were visibly broken apart, approximately 3-5 min (Beers et al., 2012). Gentle cell dissociation buffer was then replaced with 1 mL of Essential 8 to stop the reaction. Colonies were then scraped from the dish surface and broken into small aggregates of cells (roughly 50 – 200 μm in diameter). The resultant aggregates were seeded into fresh Geltrex-coated wells containing Essential 8 at split ratios of 1:5 to 1:20. iPSCs were maintained in culture for 20 weeks after thawing at which point the culture was terminated and a fresh vial of low-passage iPSCs was thawed from the liquid nitrogen. We confirmed our iPSC cell banks have normal copy number at various mutation hotspots using the hPSC Genetic Analysis Kit (Cat # 07550, STEMCELL Technologies, Vancouver, BC, Canada).

Single cell iPSC passaging was achieved using StemPro Accutase (Cat# A1110501, ThermoFisher). iPSCs were treated with Accutase at 37°C for 8-10 min and triturated to create a single cell suspension. Single cells were plated in medium supplemented with the Rho-associated kinase inhibitor (ROCKi), Y-27632 (Cat# Y-5301, LC Laboratories, Woburn, MA, United States) to promote single cell iPSC survival (Watanabe et al., 2007).

### CRISPR-Cas9 Gene Ablation

*PANX1* knockout iPSCs were created as described previously (Esseltine et al., 2020). Briefly, iPSCs were transfected using the Mirus TransIT^®^-LT1 Transfection Reagent (Cat# MIR-2304, Mirus Bio LLC, Madison, WI, United States) with the pSpCas9(BB)-2A-GFP plasmid (Cat# 48138, Addgene, Cambridge, MA, United States) according to the manufacturer’s instructions (Ran et al., 2013). The sgRNA was designed using the Sanger Institute CRISPR finder^1^ and was selected based on its low exonic off-target prediction [human *PANX1*: Sanger sgRNA ID 1087081842 (5′-GCTGCGAAACGCCAGAACAG-3′)]. After transfection, GFP-expressing single cells were sorted using fluorescence activated cell sorting (FACS) and re-plated at low density to permit easy isolation of individual clones. The resulting individual clones were examined for ablation of the target gene at the genomic level via PCR and Sanger sequencing while ablation of the PANX1 protein was assessed via immunofluorescence, Western blotting, and flow cytometry.

### Embryoid Body Generation for 3D Spontaneous Differentiation

Embryoid bodies (EBs) of 9000 cells each were created in 96-well round-bottom plates coated with 1% agarose prepared in deionized water to confer a non-adherent surface which promotes iPSC self-aggregation (Friedrich et al., 2009; Dahlmann et al., 2013). A single-cell iPSC suspension was created via Accutase dissociation as described above and re-suspended in Essential 6, which lacks the essential pluripotency factors TGFβ and FGF2 (Cat# A1516401, ThermoFisher), supplemented with 10 μM Y-27632 to promote cell survival (Lin and Chen, 2014). Essential 6 media was replenished every other day to promote spontaneous differentiation.

### Monolayer Directed Differentiation to the Three Germ Layers

Directed differentiation into the three germ layers was achieved using the STEMdiff^TM^ Trilineage Differentiation Kit (Cat# 05230, STEMCELL Technologies) according to the manufacturer’s instructions.

### Quantitative Reverse Transcription Polymerase Chain Reaction

Undifferentiated iPSCs, along with differentiated cells and embryoid bodies were collected for gene expression analysis. RNA was extracted using the PureLink^TM^ RNA isolation kit (Cat # 12183018A, ThermoFisher) with DNase I treatment according to the manufacturers’ instructions. Purified RNA was quantified using a NanoDrop^TM^ 2000 spectrophotometer (Cat# ND-2000, ThermoFisher), and stored at −80°C until use. High quality RNA was identified by a λ260/280 of ≥ 2.0 and λ260/230 of ≥ 2.0.

RNA was converted into complementary DNA (cDNA) using the High-Capacity cDNA Reverse Transcription Kit (Cat# 4368814, ThermoFisher) according to the manufacturer’s instructions. Typically, 500 ng of RNA were used per cDNA reaction. The resulting cDNA was stored at −30°C until use.

Quantitative reverse transcription polymerase chain reaction (qPCR) was performed using intercalating dye chemistry (Zipper et al., 2004). Oligonucleotide sets listed in Table 1 were designed for specific target amplification and minimal primer dimer formation using NCBI Primer-BLAST (NIH, Bethesda, MD, United States^2^) and IDT’s Oligo Analyzer Tool (IDT, Newark, NJ, United States). Bio-Rad SsoAdvanced^TM^ Universal SYBR^®^ Green Supermix (Cat# 1725274, Bio-Rad, Hercules, CA, United States) was utilized and oligonucleotides (all from IDT) were used at 10 μM in each reaction. Standard run time cycling parameters were as follows: one cycle of 50°C for 2 min, one cycle of 95°C for 30 s, 40 cycles of 95°C for 10 s, 60°C for 1 min, followed by a melt curve from 60°C to 95°C. Data was analyzed using QuantStudio^TM^ real-time PCR software (Version1.3, ThermoFisher). Gene expression for each sample were normalized to the reference gene (*GAPDH* or 18SrRNA) to generate a deltaC_*T*_ (ΔC_*T*_) (Schmittgen and Livak, 2008). Stable expression of *GAPDH* throughout differentiation was confirmed by 1-way analysis of variance (ANOVA) across all cell lines and tissue types generated in this study according to the methodology described in Schmittgen and Livak (2008) (data not shown) (Schmittgen and Livak, 2008). Samples where *C*_*T*_-values were ≥ the *C*_*T*_-value of the no-template control were considered qPCR non-detects and were excluded from further analysis.

**TABLE 1:** Oligo sets for qPCR.

Target	Forward Primer (5′-3′)	Reverse Primer (5′-3′)	Amplicon Size (bp)
***18S***	GTAACCCGTTGAACCCCATT	CCATCCAATCGGTAGTAGCG	151
***CXCR4***	AGGGGATCAGTATATACACTTCAG	AGAAGGGAAGCGTGATGACA	276
***FOXA2***	Hs.PT.58.22972176		
***GAPDH***	TGCTTTTAACTCTGGTAAAG	CACTTGATTTTGGAGGGATC	198
***GJA1***	GGTCTGAGTGCCTGAACTTGCCT	AGCCACACCTTCCCTCCAGCA	184
***HNF1B***	AGCCAGTCGGTTTTACAGCA	CTTGGGAGGTGTTGAGGCTT	229
***KDR***	GTACATAGTTGTCGTTGTAGG	TCAATCCCCACATTTAGTTC	132
***MIXL1***	GCTTTCAGTTACCCTCCCAGATAAC	GCACAGGAAGTACAATAACAAGTGC	270
***NANOG***	TGCTGAGATGCCTCACACGGA	TGACCGGGACCTTGTCTTCCTT	155
***NCAM1***	GCCTGAAGCCCGAAACAAC	CACTGGGTTCCCCTTGGA	117
***NES***	CTGCGGGCTACTGAAAAGTT	TCCAGGAGGGTCCTGTACG	161
***PANX1***	GGCAAAGGGAAAGCGAAAG	CCAGGAGAAAGAACTTGGAGAG	337
***PANX2***	CTACATCCTCGGCACCAAGA	GGGTACGGGATTTCCTTCTC	168
***PANX3***	Hs.PT.58.4636086		
***PAX6***	CAGCTCGGTGGTGTCTTTG	CCGTTGGACACGTTTTGATTG	167
***PDGFRA***	CTGCTGATGAAAGCACACGG	AACTCCATTCCTCGGGCAAC	289
***POU5F1***	TGGGCTCGAGAAGGATGTG	GCATAGTCGCTGCTTGATCG	78
***SOX17***	GAGCCAAGGGCGAGTCCCGTA	CCTTCCACGACTTGCCCAGCAT	141
***T***	Hs.PT.58.1243965		
***WT1***	GTAGCCCCGACTCTTGTACG	AGTCCTGGTGTGGGTCTTCA	297

The heatmap in Figure 5C was generated from the average 2^–Δ*CT*^ value (fold change from *GAPDH* expression) of each condition using R Studio (version 3.6.1) software with the ggplots.2 package, heatmap.2 function, and row scaling. For all other qPCR analysis, fold change expression of genes relative to a control sample (such as the control (wildtype) iPSC cell population) were evaluated using the ΔΔC_*T*_ method as described (Schmittgen and Livak, 2008). Fold change represents the standard error of the mean of 2-8 technical replicates were plotted in GraphPad PRISM (Version 6.07, GraphPad, San Diego, CA, United States).

### Immunofluorescence

Embryoid bodies and monolayer cultures were fixed in 10% buffered formalin (Cat# CA71007-344, VWR, Radnor, PA, United States) for 1 h at room temperature. Fourteen day old (day 14) EBs were cryogenically prepared and immunolabelled according to the methodology described in STEMCELL Technologies’ Document #27171, Version 1.0.0, Nov 2019. In summary, day 14 EBs were first dehydrated in Ca^2+/^Mg^2+^-free PBS supplemented with 30% sucrose for 1-4 days at 4°C until the EBs sank. Dehydrated EBs were then incubated for 1 hour at 37°C in gelatin embedding solution consisting of 10% sucrose and 7.5% gelatin prepared in Ca^2+/^Mg^2+^-free PBS. The EBs were then transferred to a cryopreservation mold and snap frozen in a slurry of dry ice and isopentane followed by cryosectioning at 14 μm slice thickness. For antigen retrieval, slides were placed into a plastic container with citrate buffer, pH 6.0: 0.294% tri-sodium citrate (dihydrate) (Cat# A12274, Alfa Aesar, Tewksbury, MA, United States) with 0.05% Tween^®^20. Samples were heated in a rice steamer for 20 minutes. Five day old (day 5) EBs were immunostained as whole-mount preparations. Immunostaining with primary antibodies, dyes, and stains indicated in Table 2 was performed as described in the figure legends. AlexaFluor-conjugated secondary antibodies were all purchased from ThermoFisher. Slides were mounted using Mowial^®^488 reagent with 1,4-diazabicyclo[2.2.2]octane (DABCO) antifade compound (Cold Spring Harbor Laboratory, 2007).

**TABLE 2:** Primary antibodies and dyes.

Marker	Supplier	Catalog #	Flow Cytometry	IF	Western Blot
**Caspase3 (active)**	BD Biosciences	559565	1:5000		
**GAPDH**	MilliporeSigma	MAB374			1:5000
**Hoechst 33342**	FisherScientific	H3570		1:10,000	
**Ki67**	Abcam	ab16667	1:1000	1:250	
**Nestin**	ThermoFisher	14-9843-82		1:500	
**OCT4**	Abcam	ab181557		1:250	
**PANX1**	Laird Lab	N/A		1:500	1:2000
**PAX6**	Abcam	ab195045		1:350	1:1000
**Phalloidin**	ThermoFisher	A34055		1:500	
**SOX17**	R&D Systems	AF1924		1:1000	1:1000
**SOX2**	R&D Systems	AF2018		1:200	
**T (Brachyury)**	Abcam	ab209665	1:400	1:1000	1:1000
**Zombie NIR^TM^**	BioLegend	423105	1:1000		

### Phase Contrast Imaging

Phase contrast images of monolayer cells and embryoid bodies were taken on a Zeiss AxioObserver microscope using 5X/0.12 NA A-Plan and 10X/0.25 NA Ph1 objectives. Images from these microscopes were taken in 8-bit greyscale using an Axiocam MRm camera and AxioVision Version 4.8.2 software. All phase contrast imaging equipment is from Carl Zeiss Microscopy (Jena, DEU).

### Confocal Microscopy

Fluorescent confocal images were acquired on an Olympus Fluoview FV10i—W3 confocal microscope (Olympus, Tokyo, JPN) fitted with a 10X/0.4, or 60X/1.2 NA lens and Fluoview version 2.1.17 software. The following lasers were used to visualize fluorophores: DAPI/Hoechst (405 nm laser); Alexa Fluor 488 (473 nm laser); Alexa Fluor 555 (559 nm laser); Alexa Fluor 647 (635 nm laser). Other images were acquired using an Olympus Fluoview FV1000 confocal microscope fitted with 10X/0.4 NA, 20X/0.75NA or 40X/0.95NA and the following lasers: 405 nm, 458 nm, 568 nm, 633 nm. Images were analyzed using FIJI open source software (Schindelin et al., 2012). To facilitate better visualization, fluorescent confocal images were occasionally subjected to uniform brightness/contrast enhancement.

### Flow Cytometry

Flow cytometry was performed on a CytoFLEX (Beckman Coulter, Brea, CA, United States) flow cytometer. Antibodies for flow cytometry were titrated over a range of concentrations prior to use. The following controls were included in all flow cytometry runs: unstained control, fluorescence-minus-one (FMO) controls, secondary-only controls, and single-color compensation controls for fluorochromes. UltraComp compensation beads (Cat# 01-2222-43, ThermoFisher) were used with antibodies raised in mice.

Live single-cell suspensions were labelled with Zombie NIR^TM^ fixable viability dye (Cat# 423105, BioLegend^®^, San Diego, CA, United States) to eliminate dead cells during the analysis stage. Next, the cells were fixed in 10% buffered formalin for 10 min at 4°C in the dark. After fixation, the cells were permeabilized (Ca^2+^Mg^2+^-free PBS with 0.5% BSA supplemented with 0.1% Triton X-100) for 15 min at room temperature in the dark. Primary and secondary antibodies (used at dilutions according to Table 2) were incubated for 30 min at 4°C in the dark. Flow cytometric analysis was performed using FlowJo software (version 10.7.1).

### SDS–PAGE and Western Blot

Cells were lysed with a solution comprising 50 mM Tris–HCl pH 8, 150 mM NaCl, 0.02% NaN_3_, 1% Triton X-100, 1 mM NaVO_4_, 10 mM NaF, 2 μg/mL leupeptin, and 2 μg/mL aprotinin. Soluble proteins were separated using SDS-PAGE and transferred to a 0.45 μm nitrocellulose membrane (Cat# 1620115, Bio-Rad). Primary antibodies (Table 2) were prepared in tris buffered saline + Tween^®^20 (TBST) + 3% skim milk and incubated overnight at 4°C. Secondary antibodies conjugated to HRP were prepared in TBST + 3% skim milk and incubated for 1 h at room temperature. Proteins were visualized with Bio-Rad Clarity^TM^ Western ECL Substrate (Cat# 1705061, Bio-Rad) using a GE ImageQuant LAS 400 (Cat# 28955810, GE Healthcare, Chicago, IL, United States).

### Statistics

Statistical analysis was performed in GraphPad PRISM Version 6.07. Error bars depict ± standard error of the mean (SEM) with *n* ≥ 3 biological replicates (independent experiments) unless otherwise stated. Statistical significance for comparisons between 2 groups was determined by unpaired Student’s *t*-test. Statistical significance for comparisons between 3 or more groups was determined by Analysis of Variance (ANOVA) followed by a Tukey’s multiple comparisons test. ^∗^*p* ≤ 0.05, ^∗∗^*p* ≤ 0.01, ^∗∗∗^*p* ≤ 0.001, ^****^p ≤ 0.0001.

## Results

### Human iPSCs Express PANX1

**FIGURE 1:**
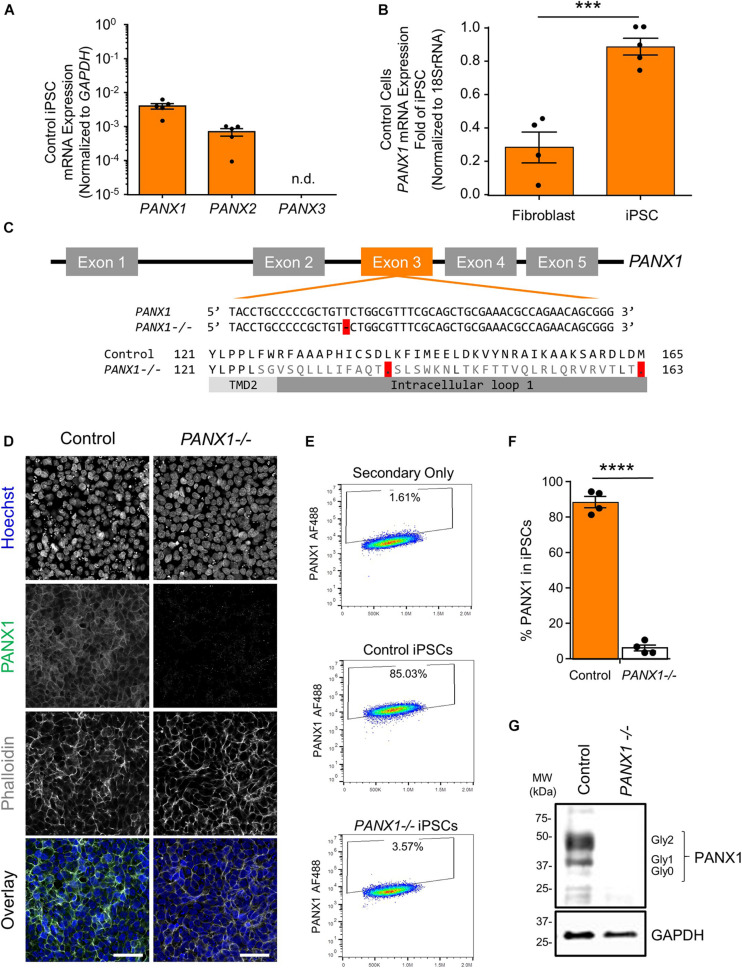
PANX1 expression and CRISPR-Cas9 gene ablation in human iPSCs. **(A)** Quantitative RT-PCR (qPCR) demonstrates the presence of *PANX1* and *PANX2* transcripts in human iPSCs, but not *PANX3*. n.d. qPCR non-detect. **(B)** *PANX1* mRNA is significantly upregulated in human iPSCs compared to the dermal fibroblasts from which they were derived. Data represent the standard error of the mean of 5 independent experiments. ***, *p* < 0.001 as assessed through Student’s *T*-test. **(C)** CRISPR-Cas9 guide RNA was designed targeting the third exon of the human *PANX1* gene. Resulting CRISPR-Cas9 engineering induced a single base pair deletion, thus disrupting the reading frame. After gene ablation, human iPSCs no longer express PANX1 protein as shown through immunofluorescence **(D)**, flow cytometry **(E,F)**, and Western blot **(G)**. In the Western blot, control human iPSCs express PANX1 protein as several discreet band sizes corresponding with the non-glycosylated protein (Gly0), high mannose (Gly1) and complex carbohydrate addition (Gly2). Immunofluorescence images were acquired using consistent parameters and are representative of more than 5 replicate experiments. Equal adjustments for brightness or contrast in FIJI were applied to both conditions. PANX1 (green); Nuclei (Hoechst, blue); Actin (Phalloidin, gray). Data represent the standard error of the mean of 4 independent experiments. ****, *p* < 0.0001. Scale bar = 50 um.

Previous research has indicated that human pluripotent stem cells express transcripts of all three pannexin family members (Hainz et al., 2018). Our qPCR analysis suggests that human iPSCs express mRNA for *PANX1* and *PANX2*, however we are unable to detect *PANX3* transcripts in these cells (Figure 1A). qPCR also reveals that *PANX1* gene expression is significantly higher in iPSCs compared to the dermal fibroblasts from which they were derived (Figure 1B). Immunofluorescence localizes PANX1 protein primarily to the cell surface of human iPSCs with lesser populations of intracellular staining (Figure 1D). Although we identified *PANX2* mRNA in our iPSCs, we were unable to find a reliable antibody to detect the PANX2 protein. Therefore, we focused our subsequent studies on PANX1.

### *PANX1-/-* iPSCs Are Morphologically Comparable to WT Control

Because *PANX1* mRNA is upregulated after iPSC reprogramming, and human *PANX1* mutations are linked to primary human oocyte death, we sought to determine whether PANX1 protein was essential for human iPSC survival, growth, or pluripotency. CRISPR-Cas9 was used to genetically ablate *PANX1* in iPSCs (Figure 1). The resulting clonal knockout iPSCs have a single base pair deletion in the third *PANX1* exon resulting in a frameshift mutation and up to 15 early stop codons within the *PANX1* transcript (Figure 1C). At the protein level, the mutation alters the amino acid sequence starting from the second transmembrane domain (Figure 1C). Western blot analysis shows PANX1 protein in control iPSCs expressed as three distinct bands relating to the three glycosylation states where Gly2 corresponds with complex carbohydrate modification, Gly1 is the addition of a high mannose species, and Gly0 PANX1 lacks glycosylation (Figure 1G; Penuela et al., 2007). After CRISPR-Cas9 gene ablation, *PANX1-/-* iPSCs no longer express PANX1 protein as shown through Western blot, flow cytometry, and immunofluorescence (Figures 1D–G).

**FIGURE 2:**
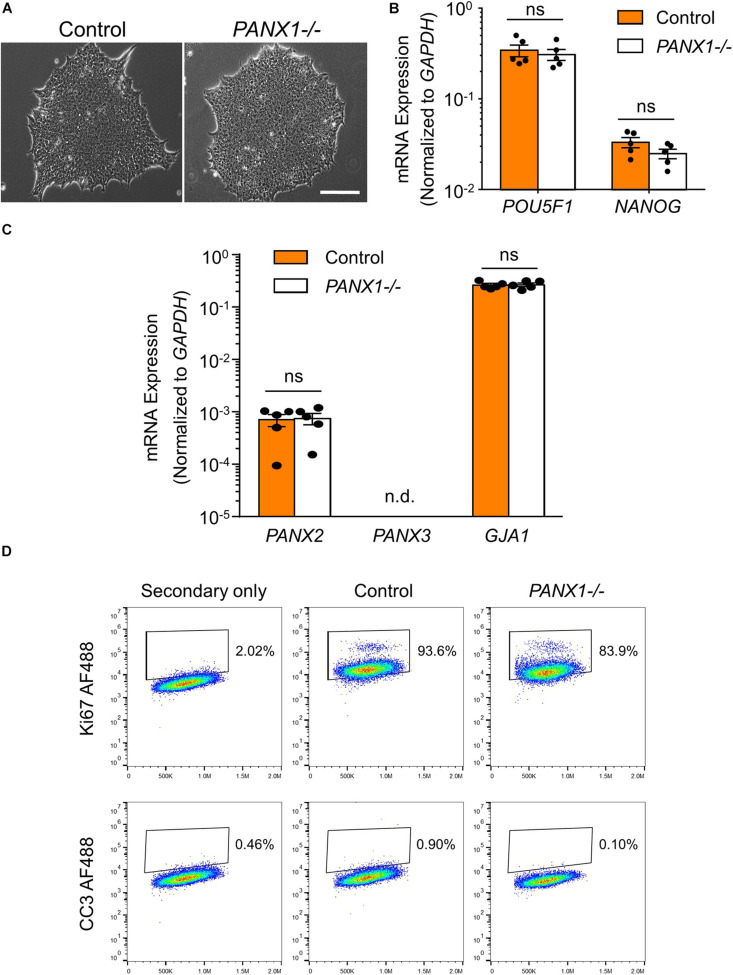
*PANX1-/-* iPSCs are morphologically comparable to control. **(A)** Phase contrast micrographs demonstrate that control and *PANX1-/-* iPSCs each grow as large colonies of tightly packed cells with refractive borders and little differentiation. Scale bar = 200 um. qPCR analysis of **(B)** the key pluripotency genes *POU5F1* (encoding for OCT4) and *NANOG* as well as **(C)** *PANX2*, *PANX3* (qPCR non-detect) or *GJA1* (encoding for Cx43) expression in control and *PANX1-/-* iPSCs. Data represent the standard error of the mean of 5 independent experiments. ns, non-significant. n.d. qPCR non-detect. **(D)** Flow cytometry evaluation of the proliferation marker Ki67 as well as the apoptotic marker cleaved caspase 3 (CC3) in control and *PANX1-/-* iPSCs.

*PANX1-/-* iPSCs appear morphologically indistinguishable from control cells and continue to grow as large colonies of tightly packed cells characteristic of human pluripotent stem cells (Figure 2A). *PANX1-/-* iPSCs continue to express similar amounts of the pluripotency markers *POU5F1* (encoding for OCT4) and *NANOG* compared to control iPSCs (Figure 2B). Furthermore, *PANX1-/-* iPSCs do not upregulate *PANX2*, *PANX3*, or *GJA1* (encoding for Cx43) in response to loss of the PANX1 protein (Figure 2C). Flow cytometry demonstrates equivalent expression of the proliferation marker, Ki67 as well as equally low expression of the apoptosis marker, cleaved caspase 3 (CC3) (Figure 2D). Therefore, *PANX1* genetic ablation does not appear to negatively impact human iPSC survival, proliferation, morphology, or pluripotency marker expression.

### PANX1 Is Alternatively Glycosylated and Differentially Localized in Cells From the Three Embryonic Germ Layers

**FIGURE 3:**
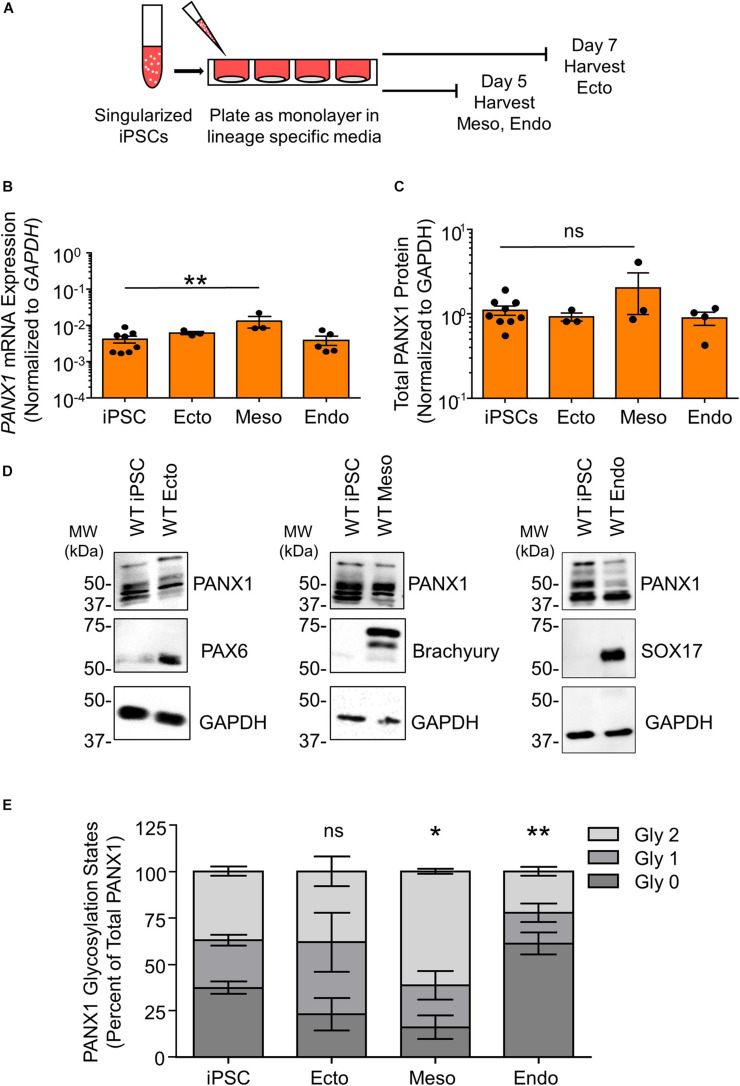
PANX1 is expressed in cells from all three embryonic germ layers. **(A)** Schematic depicting 2D directed germ lineage differentiation. **(B)** qPCR analysis of *PANX1* mRNA expression in control human iPSCs as well as after directed differentiation into each of the three embryonic germ layers, ectoderm (ecto), mesoderm (meso), and endoderm (endo). **(C)** Densitometric analysis of total PANX1 protein expression in iPSCs, ectoderm, mesoderm, and endoderm cells. **(D)** Representative Western blots and **(E)** densitometric analysis of the three PANX1 glycosylation states. Gly0, non-glycosylated protein; Gly1, high mannose; Gly2, complex carbohydrate addition. Data represent the standard error of the mean of 3-10 independent experiments. ns, no significance; *, *p* < 0.05; **, *p* < 0.01 compared to iPSCs.

Although *PANX1* genetic ablation was well tolerated in human iPSCs, we investigated whether this protein plays a role in cell fate specification. Given the wide expression of PANX1 across many tissues of the body, we examined PANX1 expression in each of the three germ layers: endoderm, ectoderm, and mesoderm (Figure 3). PANX1 was indeed expressed in each germ layer as shown through qPCR and Western blot (Figures 3B–D). *PANX1* transcript abundance was significantly elevated in mesoderm cells compared to undifferentiated iPSCs (Figure 3B). However, this increased mRNA abundance did not translate into a similar increase in PANX1 protein expression in mesoderm cells (Figures 3C,D). Similar to what was observed in iPSCs, densitometric analysis of the three glycosylation species revealed that 37.15 ± 2.47% of ectoderm PANX1 is fully glycosylated while 25.59 ± 2.91% exists as high mannose and 37.24 ± 3.28% is unglycosylated (Figures 3D,E). Interestingly, PANX1 in mesoderm cells is significantly more glycosylated than iPSCs (61.25 ± 1.29% Gly2, 22.71 ± 7.58% Gly1 and 16.01 ± 6.43% Gly0). On the other hand, endoderm cells appeared to have a significant reduction in the Gly1 and Gly2 species. Endodermal PANX1 exists as 61.09 ± 5.84% unglycosylated species while only 22.38 ± 2.35% is fully glycosylated with complex carbohydrate species (Figure 3E).

**FIGURE 4:**
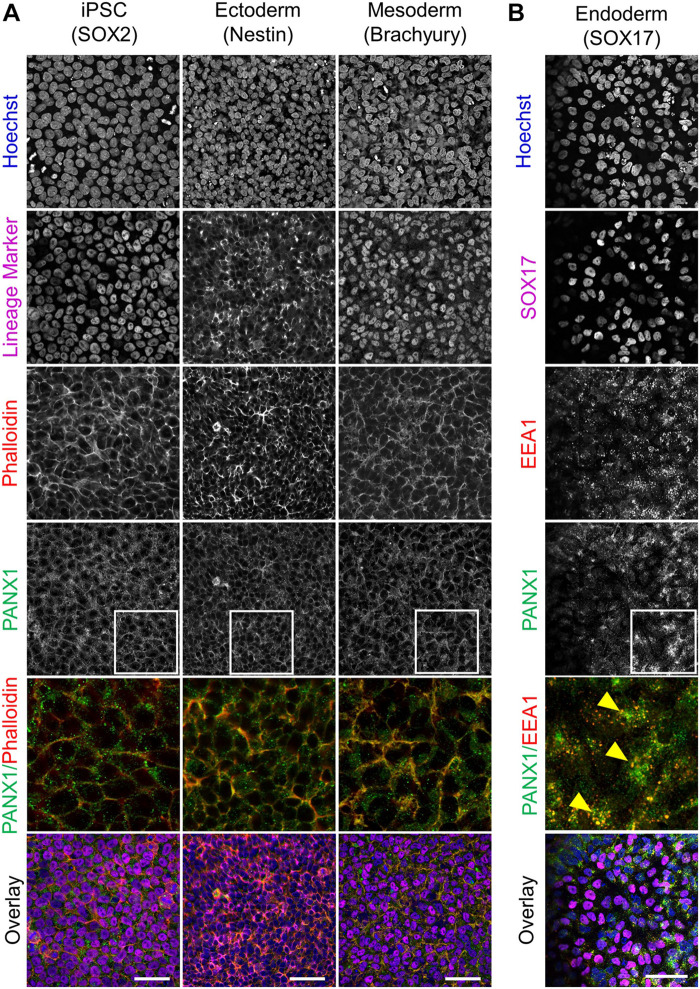
PANX1 is differentially localized in the three embryonic germ layers. **(A)** Immunofluorescent evaluation of PANX1 (green) demonstrates primarily cell surface localization in iPSCs, ectoderm and mesoderm with lesser intracellular pools. Nuclei (Hoechst, blue); lineage markers (SOX2, Nestin, Brachyury, magenta); Actin (phalloidin, red). **(B)** In endoderm cells PANX1 is localized intracellularly where it partially overlaps with early endosomes (EEA1, red; SOX17, magenta). Inset: regions of interest zoomed in to highlight regional PANX1 localization. Brightness and contrast were equally adjusted across conditions in FIJI. Scale bar = 50 μm.

Because glycosylation has been reported to play a role in PANX1 trafficking to the plasma membrane, we also evaluated the subcellular distribution of PANX1 in the three germ lineages. Immunofluorescence shows PANX1 protein localization at the cell surface in Nestin positive ectoderm cells and Brachyury positive mesoderm cells (Figure 4A, inset). However, in SOX17 positive endoderm cells, PANX1 localized mainly to intracellular compartments and overlapped with EEA1, indicating PANX1 localization in early endosomes (Figure 4B inset, yellow arrowheads).

### *PANX1* Knockout Embryoid Bodies Exhibit Skewed Lineage Specification

**FIGURE 5:**
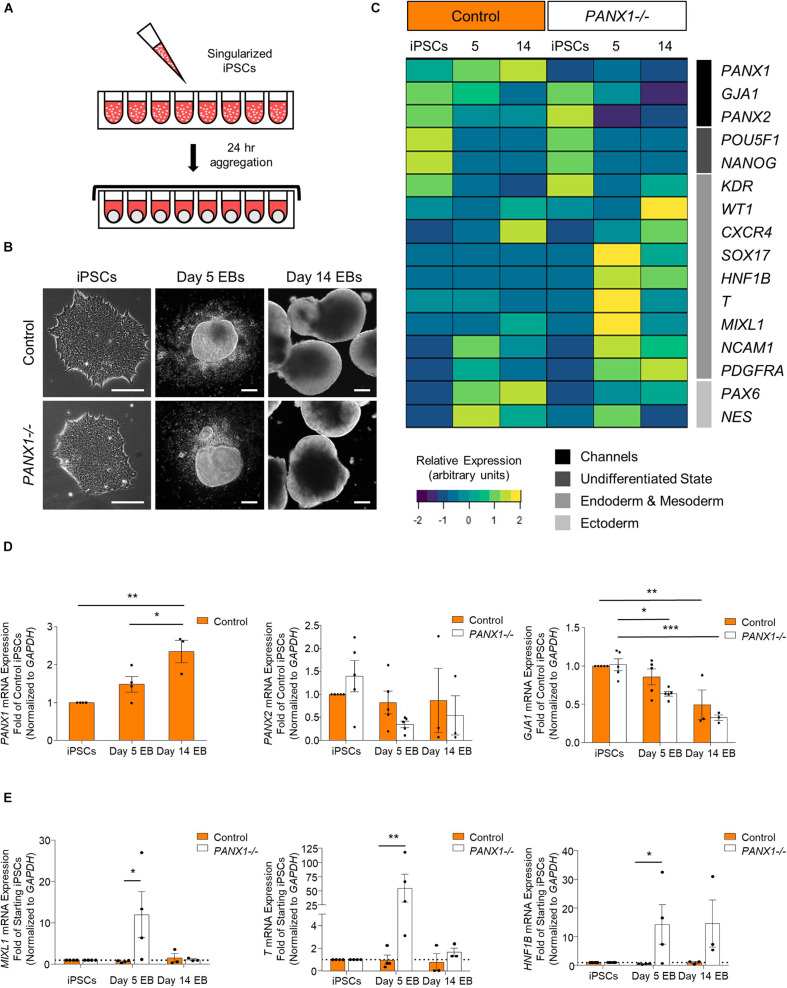
*PANX1* knockout embryoid bodies exhibit skewed lineage specification. **(A)** Schematic depicting 3D embryoid body (EB) formation. **(B)** Control and *PANX1-/-* iPSCs self-aggregate and form embryoid bodies. Scale bar = 200 um. **(C)** Gene expression analysis of control and *PANX1-/-* iPSCs as well as embryoid bodies after 5 and 14 days of spontaneous differentiation. The heatmap was generated using the average 2^–Δ*CT*^ data value (normalized to *GAPDH*) for each condition using R Studio software with the ggplots. 2 package and row clustering. Transcripts for *PANX3* were not detected at any point during differentiation. **(D)** *PANX1*, *PANX2* and *GJA1* (Cx43) qPCR gene expression analysis of control and *PANX1-/-* iPSCs and embryoid bodies after 5 and 14 days of spontaneous differentiation. **(E)** Lineage-specific gene expression of control and *PANX1-/-* iPSCs and embryoid bodies after 5 and 14 days of spontaneous differentiation. Bar graph data presented as 2^–ΔΔ*CT*^ (fold change of iPSCs). Data represents the mean of 3-5 independent experiments. ^∗^, *p* < 0.05; ^∗∗^, *p* < 0.001; ^∗∗∗^, *p* < 0.0001 compared to starting iPSCs.

Spontaneous differentiation enables passive, cell-guided cell fate specification and generally results in cell populations from all three germ layers (ectoderm, endoderm, and mesoderm). In order to determine whether loss of PANX1 altered inherent lineage specification of human iPSCs, we investigated the spontaneous differentiation potential of control and *PANX1-/-* iPSCs cultured as 3-dimensional embryoid bodies (EBs) (Figure 5). Control and *PANX1-/-* iPSCs self-aggregated into embryoid bodies of comparable size and shape after 24 h in culture (Figure 5B). As expected, both control and *PANX1-/-* EBs downregulated genes associated with undifferentiated state (*POU5F1* and *NANOG*) relative to starting iPSCs, indicating that the cells within the EBs were losing pluripotent stemness and were committing to downstream lineages (Figure 5C). *PANX1* mRNA expression significantly increased in spontaneously differentiated EBs compared to undifferentiated iPSCs (Figure 5D) while *PANX2* mRNA remained constant throughout differentiation in both control and *PANX1-/-* EBs. On the other hand, *GJA1* (Cx43) mRNA significantly decreased throughout EB differentiation, although there was no difference in *GJA1* expression between control and *PANX1-/-* EBs (Figure 5D).

Comprehensive gene expression analysis shows altered expression of genes associated with the germ layers in *PANX1-/-* EBs compared to control (Figures 5C,E). Indeed, after 5 days of spontaneous differentiation, expression of mesendoderm (*MIXL1*), mesoderm (*T*, *PDGFRA*, *NCAM1*) and endoderm (*SOX17*, *HNF1B*) markers were elevated in *PANX1-/-* EBs compared to control (Figures 5C,E). Time course analysis suggests that at 5 days of spontaneous differentiation, expression of endoderm and mesoderm lineage genes are higher in *PANX1-/-* EBs compared to control, and decline by day 14 (Figure 5E) consistent with mesendoderm commitment and subsequent downstream lineage progression.

**FIGURE 6:**
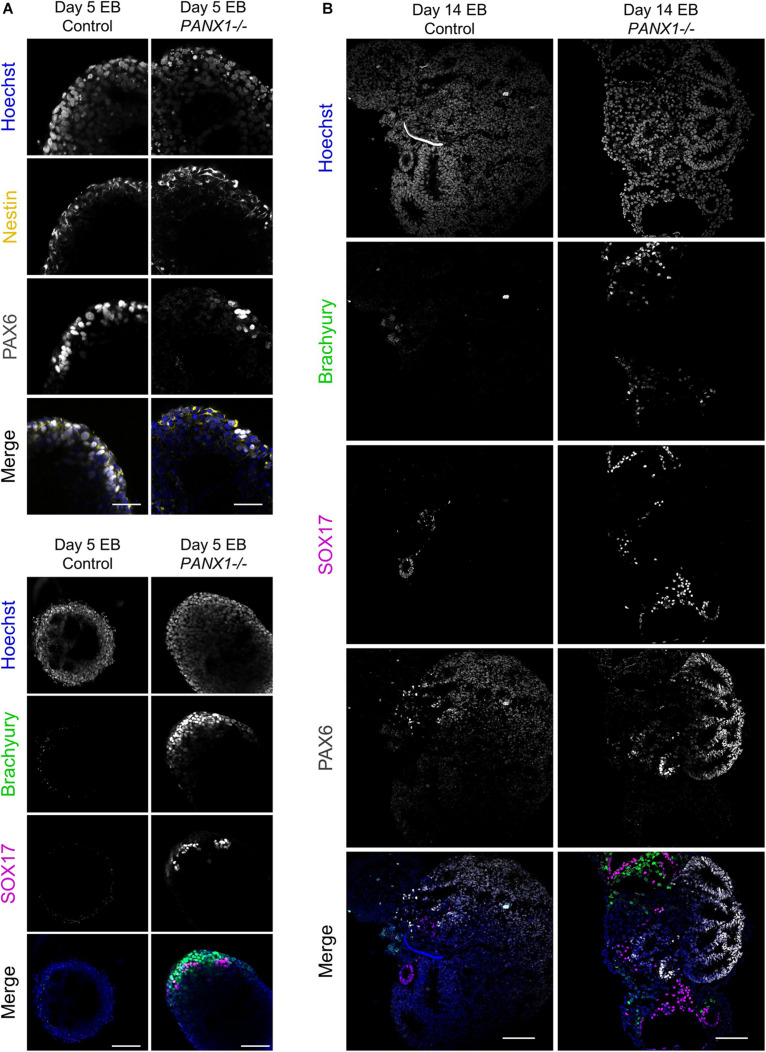
Immunofluorescent analysis of *PANX1-/-* embryoid bodies. **(A)** Immunofluorescence of ectoderm (Nestin, yellow; PAX6, gray), mesoderm (Brachyury, green) and endoderm (SOX17, magenta) in control and *PANX1-/-* day 5 whole mount EBs. **(B)** After 14 days of differentiation, control and *PANX1-/-* EBs were cryosectioned and evaluated for germ lineage expression. Equal brightness contrast enhancements were made in FIJI for picture clarity. Nuclei (Hoechst, blue). Scale bar = 100 μm.

We corroborated our qPCR data using immunofluorescence imaging of control and *PANX1-/-* embryoid bodies at day 5 (Figure 6A) and day 14 (Figure 6B). Consistent with our gene expression analysis, we observed a greater proportion of *PANX1-/-* cells expressing Brachyury (mesoderm) and SOX17 (endoderm) relative to control (Figure 6). On the other hand, both control and *PANX1-/-* EBs expressed the ectoderm markers PAX6 and Nestin (Figure 6). Taken together, spontaneously differentiated *PANX1-/-* EBs appear to favor formation of mesoderm and endoderm germ layers while the capacity to form ectodermal cells is minimally impacted.

### Exogenous Pressures Override *PANX1-/-* Lineage Bias

**FIGURE 7:**
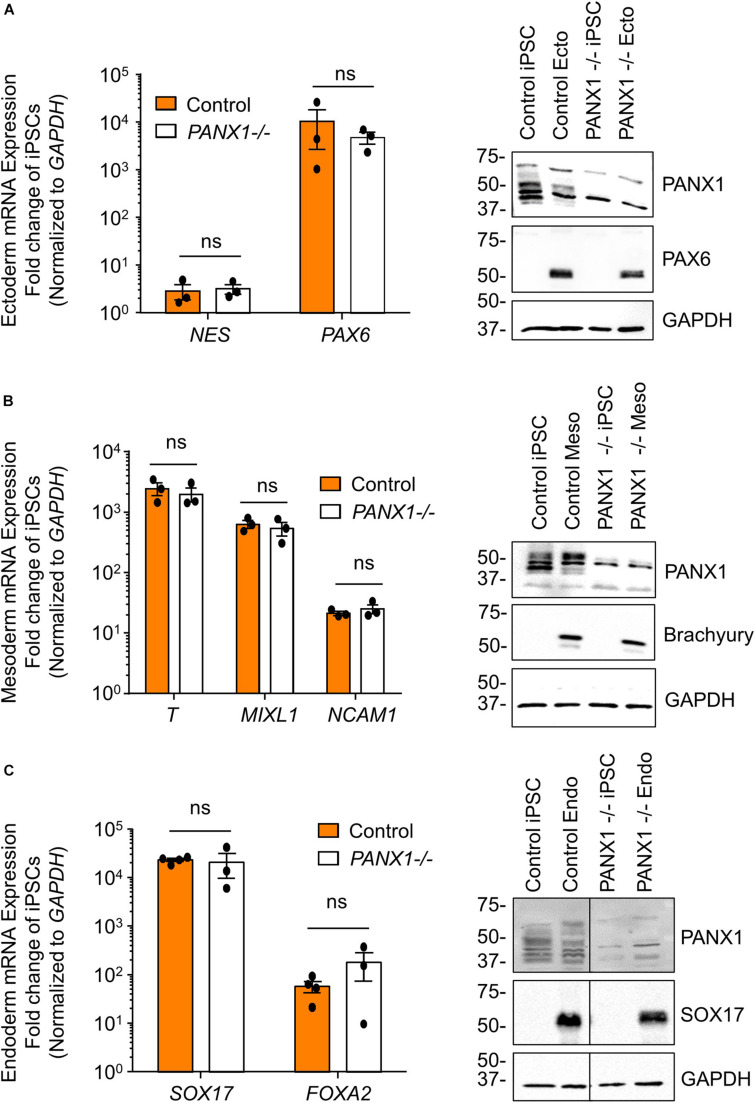
Exogenous pressures override *PANX1-/-* lineage bias. qPCR gene expression analysis and Western blot of lineage-specific markers after directed differentiation of control and *PANX1-/-* iPSCs into **(A)** ectoderm (NES, PAX6), **(B)** mesoderm (T (Brachyury), MIXL1, NCAM1) and **(C)** endoderm (SOX17, FOXA2). Data represent the standard error of the mean of 3-4 independent experiments. ns, non-significant compared to control.

**FIGURE 8:**
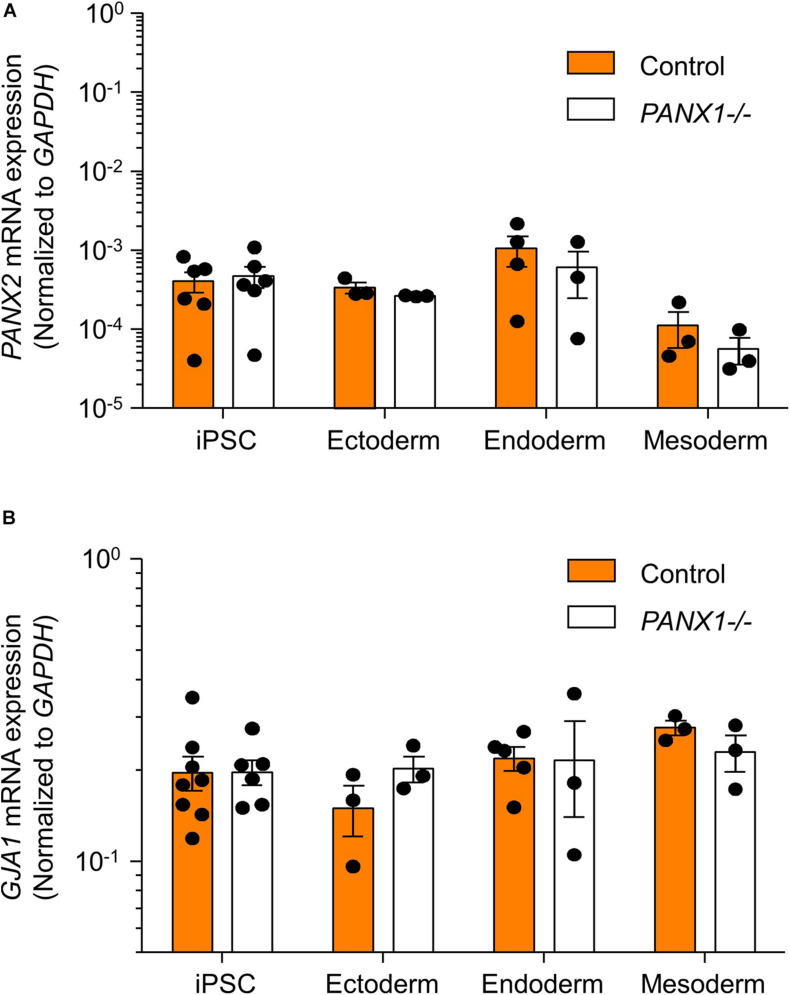
PANX2 and Cx43 do not compensate for PANX1 loss during germ lineage differentiation. qPCR analysis of **(A)** *PANX2* and **(B)** *GJA1* (Cx43) mRNA expression in directed ectoderm, mesoderm, and endoderm cultures. Data presented as 2^– Δ*CT*^ (fold change from *GAPDH*) and represent the standard error of the mean of 3-8 independent experiments.

We determined above that *PANX1-/-* iPSCs exhibit apparent lineage specification bias when permitted to spontaneously differentiate. However, we also wanted to determine whether loss of PANX1 impacted directed germ lineage differentiation promoted through the application of exogenous growth factors and small molecules. To that end, we used the STEMdiff^TM^ Trilineage Differentiation Kit (STEMCELL Technologies) to evaluate the ability of *PANX1-/-* iPSCs to differentiate into cells from all three germ layers in response to external pressures. Despite the demonstrated lineage biases outlined above in the passive embryoid body cultures, both qPCR and Western blot demonstrate that *PANX1-/-* iPSCs effectively differentiate into cells of all three germ lineages when cultured with the Trilineage Differentiation Kit (Figure 7). Similar to what we observed in the undifferentiated iPSCs, both *PANX2* and *GJA1* (Cx43) transcripts are expressed in the three germ layers but are not upregulated in compensation for the loss of PANX1 during directed differentiation (Figure 8). Furthermore, *PANX3* transcripts remained undetectable by qPCR in cells from all three germ layers (data not shown). Therefore, although we find that PANX1 influences germ layer specification, it is not essential to this process.

## Discussion

Here we find that PANX1 protein is expressed in human iPSCs as well as in cells of all three embryonic germ lineages: ectoderm, endoderm, and mesoderm, further underlying the potential for this protein to participate in cell fate specification. Despite being dispensable for stem cell morphology, proliferation, and survival, PANX1 does contribute to cell fate specification with exaggerated mesendoderm cell abundance in spontaneously differentiated *PANX1-/-* cultures compared to control. Having confirmed *PANX1* gene ablation through genotyping, immunofluorescence, Western blot, flow cytometry and qPCR, we are confident that our clonal knockout cells are indeed deficient in PANX1 protein. However, we do occasionally detect non-specific bands in our PANX1 Western blots. Depending on PANX1 expression and total protein loaded onto gels, we have found that these non-specific bands generally appear when longer exposure times were necessary to capture sufficient PANX1 signal. However, these bands were consistent across different samples and thus considered non-specific.

The human protein atlas reports that PANX1 is widely expressed in tissues arising from all three germ layers^3^ and the many mouse studies conducted around the world have highlighted how important murine PANX1 is in postnatal health and disease. For example, PANX1 is reported to exacerbate the spread of ischemic injury in mice following stroke via the disruption of electrochemical gradients in neurons and glial cell types (Chekeni et al., 2010; Bond and Naus, 2014). Human PANX1 also has reported involvement in pathogen-mediated activation of the caspase cascade by releasing ATP which attracts phagocytic cells, resulting in the clearance of the damaged/infected cells (Chekeni et al., 2010; Bond and Naus, 2014). On the other hand, HIV can use the PANX1 channel to enter lymphocytes (Orellana et al., 2013) and once inside the cell, the virus can elicit PANX1-mediated ATP release to destabilize the cell membrane and ultimately facilitate viral spread (Séror et al., 2011; Penuela et al., 2014). Surprisingly, few studies have focused on human pannexin proteins or the role of pannexins in early development. We are now able to uncover how PANX1 signaling influences the earliest developmental decisions through spontaneous and directed differentiation of human iPSCs, and by modeling human tissue development using PSC-derived organoids.

PANX1 is expressed in the human oocyte as well as the 2- and 4-cell stage embryo (Sang et al., 2019). Human embryonic stem cells and induced pluripotent stem cells have previously been shown to express all three *PANX* transcripts (Hainz et al., 2018). However, we only detect expression of *PANX1* and *PANX2* and were unable to detect *PANX3* in any of the stem cell types that we evaluated. We also show that human iPSCs express PANX1 protein, which is concentrated at the cell surface of undifferentiated iPSCs with lesser amounts of intracellular PANX1 populations. Furthermore, PANX1 expression persists in cells of all three embryonic germ layers. Interestingly, while PANX1 was primarily at the cell surface of iPSCs, ectoderm and mesoderm cells, it was redistributed to intracellular compartments in endoderm cells where it partially overlapped with early endosomes. Prolonged increases in ATP can induce PANX1 internalization to endosomal compartments (Boyce et al., 2015). It remains unclear whether a similar mechanism triggers PANX1 internalization during endoderm differentiation. Intracellular PANX1 localization has been shown to correlate with reduced PANX1 glycosylation. We see a similar trend here, with PANX1 in endoderm cells exhibiting both decreased Gly1 and Gly2 and corresponding intracellular localization. However, in the absence of glycosylation mutant studies, we are unable to say definitively that the reduction in PANX1 glycosylation drives intracellular redistribution. It remains to be seen what role this intracellular PANX1 pool plays in endodermal tissues, but given that our *PANX1-/-* iPSCs exhibit enhanced endodermal differentiation, removing this protein is clearly beneficial for endodermal lineage commitment. One might suppose that because endoderm cells internalize PANX1 they do not need a functional PANX1 pool and might therefore benefit from *PANX1* knockout. However, the same cannot be said for the mesoderm cells, which retain PANX1 at the cell surface, and this germ lineage is equally enhanced in *PANX1-/-* iPSCs.

Most of what is currently understood about pannexins in stem cell fate specification arises from somatic (adult or tissue-resident) stem cell studies. Pannexin signaling can influence the self-renewal and differentiation of multiple somatic stem cell types including osteoprogenitor cells, skeletal myoblasts, and postnatal neuronal stem cells (Swayne and Bennett, 2016; Ishikawa and Yamada, 2017; Pham et al., 2018). PANX1 is implicated in neural precursor cell maintenance and proliferation, while neuronal differentiation involves both PANX1 and PANX2 (reviewed in Swayne and Bennett, 2016). PANX1 specifically regulates neurite growth and dendritic spine density in mice whereas PANX2 influences retinoic acid-induced neurite extension (Swayne et al., 2010; Wicki-Stordeur et al., 2012, 2016; Wicki-Stordeur and Swayne, 2013; Horton et al., 2017; Xu et al., 2018). Meanwhile, PANX1 and PANX3 are both expressed in mesodermal tissues such as bone and cartilage and they have both been implicated in the regulation and commitment of resident progenitor cell populations in mice (Bond et al., 2011; Ishikawa and Yamada, 2017; Lee et al., 2018; Pham et al., 2018). PANX1 is expressed in murine bone marrow-derived stromal cells while PANX3 inhibits osteoprogenitor cell proliferation and contributes to chondrocyte differentiation (Bond et al., 2011; Ishikawa et al., 2014). In contrast to these studies on adult progenitor cell populations, very little is understood about pannexins in pluripotent stem cells, including mouse and human ESCs or iPSCs.

Human germline *PANX1* mutations which cause decreased protein abundance and trafficking defects lead to human oocyte death and female infertility (Sang et al., 2019). This effect can be mimicked using isolated mouse oocytes injected with *PANX1* mutant complementary RNA (cRNA). On the other hand, *PANX1* knockout mice remain fertile and continue to birth average litter sizes. These observed differences in fecundity may be due to inherent species differences, or *in vitro* versus *in vivo* manipulations. We find that CRISPR-Cas9 genetic ablation of *PANX1* does not negatively impact human iPSC proliferation, survival, or morphology. It is possible that *PANX1* missense mutations are more impactful than complete ablation due to as-yet unknown gain of function properties or changes in protein partner interactions. It would be interesting to examine whether human iPSCs are amenable to insertion of human missense mutations via gene editing or whether *PANX1-/-* iPSCs can differentiate to primordial germ cells.

The most well-defined role of PANX1 is in the regulated release of ATP through several mechanisms including mechanical stress, membrane depolarization, changes in intracellular ion concentration and others (Penuela et al., 2013). Autocrine and paracrine signaling mechanisms triggered through cellular release of ATP and ADP have reported trophic, differentiating, and immunomodulatory effects and ATP signaling has been linked to proliferation of mouse embryonic stem cell and several postnatal progenitor cell populations (Heo and Han, 2006; Cavaliere et al., 2015). Activated pannexin channels appear to play a supporting role in augmenting purinergic receptor activity through the release of extracellular nucleotides and nucleosides. Additionally, PANX1 has been widely implicated in cell death signaling (Chekeni et al., 2010; Penuela et al., 2014; Imamura et al., 2020). Apoptotic induction through caspase activation leads to cleavage of the PANX1 carboxyl-terminal tail, which subsequently results in release of ATP and other small molecules into the extracellular space. Although we have found that iPSCs tolerate the loss of PANX1, follow up investigations are necessary to determine whether the lineage bias that we have presented here actually results from compromised ectodermal lineage specification or due to altered proliferation or apoptosis of certain germ layers within *PANX1* null cells. The effect of PANX1 on lineage specification may in fact be combinatorial as we see increase mesendoderm gene expression while others have reported selective loss of multipotent ectodermal progenitor cells in *PANX1* knockout mice (Wicki-Stordeur et al., 2016).

Human developmental disorders arise in part due to flawed cell fate specification which can contribute to organ and tissue dysfunction. Until we have a comprehensive understanding of human development and cell fate decisions our capacity to treat developmental disorders remains incomplete. Given the ubiquitous expression of PANX1 in adult tissues, we expect one or more aspects of human stem cell pluripotency or early lineage specification to be affected by the loss of PANX1. Future studies will uncover protein interacting partners and specific messenger molecules involved in this process. Furthermore, it will be interesting to determine whether PANX1 is similarly involved in downstream specification of various terminally differentiated cells or 3-dimensional organoid development.

## Data Availability Statement

The raw data supporting the conclusions of this article will be made available by the authors, without undue reservation.

## Ethics Statement

The studies involving human participants were reviewed and approved by Newfoundland and Labrador Human Research Ethics Board #2018.210. Written informed consent for participation was not required for this study in accordance with the national legislation and the institutional requirements.

## Author Contributions

JE used CRISPR/Cas9 to generate the *PANX1-/-* iPSCs and oversaw the project and wrote the manuscript. RN and GC performed all the experiments, analyzed the data, and assembled the figures. All authors reviewed and edited the manuscript.

## Conflict of Interest

The authors declare that the research was conducted in the absence of any commercial or financial relationships that could be construed as a potential conflict of interest.

## Footnotes

1 http://www.sanger.ac.uk/htgt/wge/

2 https://www.ncbi.nlm.nih.gov/tools/primer-blast/

3 https://www.proteinatlas.org/ENSG00000110218-PANX1/tissue
